# First Complete Genome Sequence of an Emerging Cucumber Green Mottle Mosaic Virus Isolate in North America

**DOI:** 10.1128/genomeA.00452-15

**Published:** 2015-05-07

**Authors:** Rugang Li, Yi Zheng, Zhangjun Fei, Kai-Shu Ling

**Affiliations:** a U.S. Department of Agriculture-Agricultural Research Service, U.S. Vegetable Laboratory, Charleston, South Carolina, USA; b Boyce Thompson Institute for Plant Research, Cornell University, Ithaca, New York, USA; c U.S. Department of Agriculture-Agricultural Research Service, Robert W. Holley Center for Agriculture and Health, Ithaca, New York, USA

## Abstract

The complete genome sequence (6,423 nucleotides [nt]) of an emerging cucumber green mottle mosaic virus (CGMMV) isolate on cucumber in North America was determined through deep sequencing of small (sRNA) and rapid amplification of cDNA ends. The virus shares 99% nucleotide sequence identity with the Asian genotype but only 90% with the European genotype.

## GENOME ANNOUNCEMENT

The cucumber green mottle mosaic virus (CGMMV) belongs to the genus *Tobamovirus* in the family *Virgaviridae* (1). CGMMV infects various cucurbit crops and has long been known in Europe (2–4), Asia (5–7), and the Middle East (8, 9). Since 2013, CGMMV was also identified as an emerging virus in greenhouse cucumber in Canada (10) and in field melon in California, USA (11). Currently, a total of 30 complete genome sequences of CGMMV are available in GenBank. Like other tobamoviruses, CGMMV has a single-stranded positive sense RNA genome with a 3′ tRNA-like structure instead of a poly(A) tail (12). The genomes of tobamoviruses encode four polypeptides, including a 124- to 132-kDa protein, a 181- to 189-kDa read-through protein, a 28- to 31-kDa movement protein (MP), and a 17- to 18-kDa coat protein (CP). Both large proteins are translated from the 5′ proximal start codon. The two small proteins are expressed from individual 3′ co-terminal subgenomic mRNAs. In this report, the complete genomic sequence of the emerging CGMMV isolate ABCA13-01 from Canada (10) was obtained. The virus infectivity was confirmed through mechanical inoculation on seedling plants of several cucurbit species, including *Cucumis sativus, Cucumis metulifer*, *Cucumis melo*, and *Citrullus lanatus*, with typical green mottle mosaic symptoms developed on systemic leaves. Symptomatic leaves were collected for total RNA isolation using the TRIzol reagent (Invitrogen, USA). The small RNA (sRNA) was separated from the total RNA by polyacrylamide gel electrophoresis, and the sRNA library was constructed according to the published protocol (13) and sequenced on an Illumina HiSeq 2000 system. The sRNA sequences were assembled into contigs using a bioinformatics pipeline (14). A single contig covering a full CGMMV genome was obtained. To identify the exact terminal sequences, the 5′ terminal end sequence was determined using rapid amplification of cDNA ends (RACE) technology (Ambion, USA). The 3′ terminal sequence was determined through ligation of a microRNA linker (rApp-CTGTAGGCACCATCAAT-amine; New England Biolabs) to the total RNA, followed by reverse transcription-PCR with a virus-specific forward primer (5′-CAGAGGCTACCACCTCGAAA-3′) and a reverse primer (5′-ATTGATGGTGCCTACAG-3′). The 5′ terminal sequences in five clones and the 3′ terminal sequences in two other clones were obtained and analyzed using the DNASTAR Lasergene 10 program (Madison, WI). A BLASTn search using the full genome sequence indicated that the Canadian CGMMV isolate showed strong (90% to 99%) nucleotide sequence identities with known CGMMV sequences. Phylogenetic analysis using the neighbor-joining program indicated that the Canadian CGMMV isolate was closely related (98% to 99% nucleotide sequence identities) to isolates with Asian origin, including China (GenBank accession no. KJ658958), India (DQ767631), Israel (KF155229, KF155230), Japan (D12505), Korea (AF417243), and Taiwan (HQ692886) but only distantly related (90%) to isolates with European origin, including Spain (GQ411361) and Russia (FJ848666, GQ495274, GQ495275). Such strong genetic relationship to the Asian isolates suggested that the Canadian CGMMV isolate may have been introduced through a contaminated cucumber seed lot that was produced in Asia. To our knowledge, this is the first complete genome sequence of CGMMV from North America.

### Nucleotide sequence accession number. 

The full genomic sequence of CGMMV isolate ABCA13-01 was deposited in GenBank under the accession no. KP772568.
